# Precisely Determining Ultralow level UO_2_^2+^ in Natural Water with Plasmonic Nanowire Interstice Sensor

**DOI:** 10.1038/srep19646

**Published:** 2016-01-21

**Authors:** Raekeun Gwak, Hongki Kim, Seung Min Yoo, Sang Yup Lee, Gyoung-Ja Lee, Min-Ku Lee, Chang-Kyu Rhee, Taejoon Kang, Bongsoo Kim

**Affiliations:** 1 KAIST, Department of Chemistry, Daejeon 34141, Korea; 2 KAIST, Department of Chemical and Biomolecular Engineering, Daejeon 34141, Korea; 3 KAERI, Nuclear Materials Development Division, Daejeon 34057, Korea; 4 KRIBB, BioNanotechnology Research Center and BioNano Health Guard Research Center, Daejeon 34141, Korea; 5 UST, Major of Nanobiotechnology and Bioinformatics, Daejeon 34113, Korea

## Abstract

Uranium is an essential raw material in nuclear energy generation; however, its use raises concerns about the possibility of severe damage to human health and the natural environment. In this work, we report an ultrasensitive uranyl ion (UO_2_^2+^) detection method in natural water that uses a plasmonic nanowire interstice (PNI) sensor combined with a DNAzyme-cleaved reaction. UO_2_^2+^ induces the cleavage of DNAzymes into enzyme strands and released strands, which include Raman-active molecules. A PNI sensor can capture the released strands, providing strong surface-enhanced Raman scattering signal. The combination of a PNI sensor and a DNAzyme-cleaved reaction significantly improves the UO_2_^2+^ detection performance, resulting in a detection limit of 1 pM and high selectivity. More importantly, the PNI sensor operates perfectly, even in UO_2_^2+^-contaminated natural water samples. This suggests the potential usefulness of a PNI sensor in practical UO_2_^2+^-sensing applications. We anticipate that diverse toxic metal ions can be detected by applying various ion-specific DNA-based ligands to PNI sensors.

Uranium, a representative radioactive metal, has been used as the main source of nuclear power generation via nuclear fission^1^. Uranium can exist in various forms in the environment, such as uranyl fluoride (UO_2_F_2_), uranyl tetrafluoride (UF_4_), uranium dioxide (UO_2_), and triuranium octoxide (U_3_O_8_). The accumulation of these compounds in the human body can lead to severe health problems^2^. For example, UO_2_F_2_ and UF_4_ cause kidney damage, and UO_2_ and U_3_O_8_ can cause certain cancers and mutations by accumulating in the lungs^3^. Uranium is also found in diverse forms in aqueous conditions. The uranyl ion (UO_2_^2+^) is the most soluble and common form^4^,^5^. Because UO_2_^2+^ can disturb organ function by accumulating in the skeleton, kidneys, lungs, and liver^6^,^7^, the detection of UO_2_^2+^ in natural water is very important.

Traditionally, UO_2_^2+^ has been detected using various physical and chemical techniques, including inductively coupled plasma-mass spectrometry^8^, radio spectrometry^9^, atomic adsorption spectrometry^10^, and phosphorimetry^11^. However, these methods present several drawbacks in that they require expensive instruments, involve complicated methods, and are time-consuming. Recently, nanomaterial-based UO_2_^2+^ sensing methods have been developed by employing fluorescence^12^,^13^, electrochemistry^14^, resonance scattering^15^,^16^, colorimetry^17^, magnetoelasticity^18^, and surface-enhanced Raman scattering (SERS)^19^,^20^,^21^. Among these techniques, SERS offers highly sensitive molecular detection because SERS signals can be obtained from a small number of molecules or even a single molecule located within a sub-10 nm metallic nano-gap (hot spot)^22^,^23^,^24^,^25^,^26^,^27^,^28^,^29^,^30^,^31^,^32^,^33^,^34^,^35^,^36^,^37^,^38^,^39^,^40^,^41^,^42^. This technique also provides molecular fingerprints and causes less photobleaching^43^,^44^,^45^,^46^,^47^,^48^. Nevertheless, there are only a few reported SERS sensors for UO_2_^2+^ detection, and most of them have not been validated in real environments. Therefore, further improvements in sensitivity, selectivity, and reproducibility are still needed for the practical sensing of UO_2_^2+^ in a real environment using a SERS sensor.

Here, we report an ultrasensitive UO_2_^2+^ detection method using a plasmonic nanowire (NW) interstice (PNI) sensor combined with a DNAzyme-cleaved reaction. SERS-based sensing methods are often affected by uncontrolled aggregation and size distribution of nanomaterials, which are detrimental to the reproducibility of the sensor^49^,^50^,^51^. A PNI platform composed of a single-crystalline Au NW with an atomically flat surface has shown improved sensitivity and reproducibility^31^. By combining the DNAzyme cleaving method with a PNI platform, we quantitatively detected UO_2_^2+^ with an ultralow detection limit of 1 pM and high selectivity. More importantly, the precise detection of UO_2_^2+^ was demonstrated in several UO_2_^2+^-contaminated natural water samples, showing the practical applicability of the PNI sensor.

## Results and Discussion

**Figure 1:**
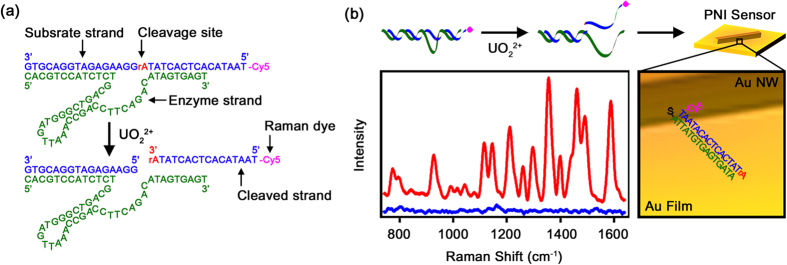
(**a**) Schematic illustration of UO_2_^2+^-specific DNAzyme and cleavage of DNAzyme induced by UO_2_^2+^ (**b**) Schematic illustration of UO_2_^2+^ detection by a PNI sensor combined with a DNAzyme-cleaved reaction. The left panel shows the SERS spectra measured from PNI sensors in the absence of (blue spectrum) and the presence of 10 nM UO_2_^2+^ (red spectrum).

DNA-based ligands, including aptamers and DNAzymes, have been widely used to detect various metal ions because they provide several advantages, such as high affinity, selectivity, stability, and relative ease of modification^52^,^53^. In this experiment, we used a DNAzyme, which has the sequence shown in Fig. 1a, for the detection of UO_2_^2+^. A DNAzyme is composed of an enzyme strand and a substrate strand. The enzyme strand (green) is a specific sequence that can bind to UO_2_^2+^. The substrate strand (blue) is hybridized with the enzyme strand. In the middle of the substrate strand, a ribose-adenosine (rA) sequence (red) is present. Ribonucleotides are approximately 100,000-fold more susceptible to hydrolytic cleavage than deoxyribonucleotides^13^. At the 5′ end of the substrate strand, Cy5 (magenta) is present. Cy5 is a well-known Raman reporter for the 633 nm excitation source^27^,^28^,^29^,^30^,^31^,^32^. When UO_2_^2+^ was added to DNAzyme, UO_2_^2+^ bound to the enzyme strand, and thus, the rA sequence on the substrate strand was hydrolytically cleaved. The cleaved strand was released from the DNAzyme and then captured by a PNI sensor. The PNI sensor was prepared by laying down a single-crystalline Au NW onto a Au film^24^ (Fig. 1b). Au NWs were synthesized using a previously reported vapor transport method^54^ and then modified by the addition of a complementary capture DNA strand to the cleaved strand. The length of capture strand was set as 15 base pairs to increase the hybridization efficiency with cleaved strand^55^. The PNI sensor with an attached cleaved strand can provide a strong Cy5 SERS signal, enabling the detection of UO_2_^2+^. The left panel in Fig. 1b shows the result of using a PNI sensor combined with a DNAzyme-cleaved reaction for UO_2_^2+^ detection. When UO_2_^2+^ was present in the sample solution at the concentration of 10 nM, 4 major bands at 1185, 1360, 1485, and 1580 cm^−1^ were clearly detected by the PNI sensor (red spectrum). These bands correspond to the *v*(C-N)_stretch_, *v*(C = C)_ring_, *v*(C-C)_ring_, and *v*(C = N)_stretch_ of Cy5, respectively^28^. In contrast, when UO_2_^2+^ was absent from the sample solution, the PNI sensor exhibited a weak SERS signal (blue spectrum). In this way, UO_2_^2+^ can be detected from the turn-on of the SERS signal. Generally, turn-on type sensors are more suitable than turn-off type sensors for practical applications because they result in fewer false-positive results^17^.

**Figure 2:**
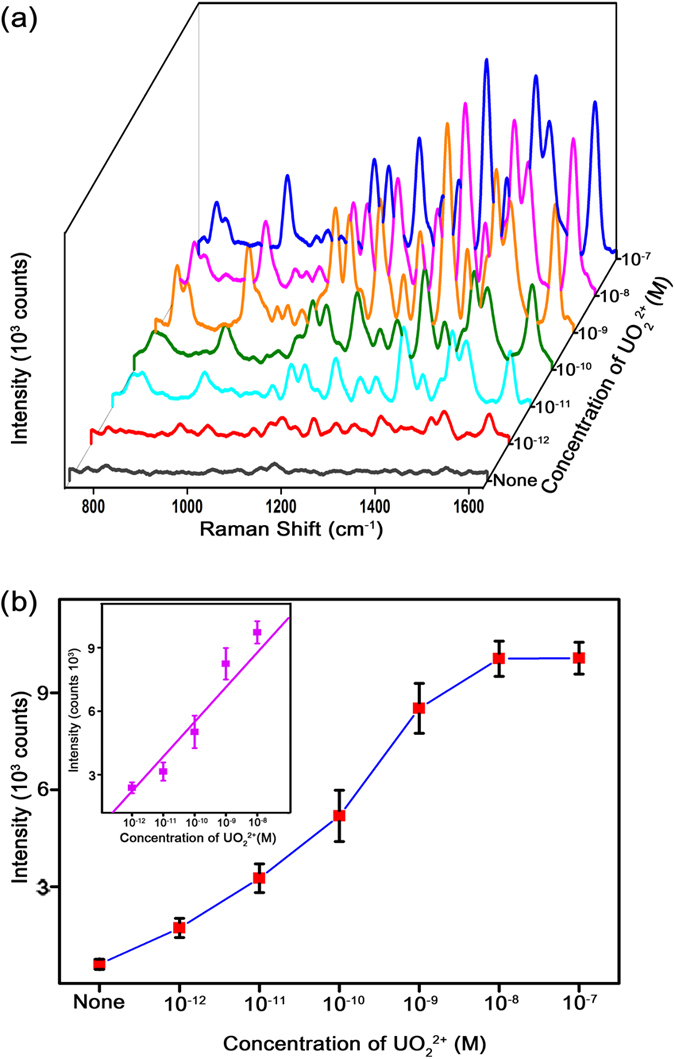
(**a**) SERS spectra of Cy5 measured from PNI sensors when the UO_2_^2+^ concentration is varied from 1 pM to 100 nM. (**b**) Intensity of the Cy5 1580 cm^−1^ band plotted as a function of the UO_2_^2+^ concentration. The inset provides the dynamic range of the PNI sensor for UO_2_^2+^ detection. The data represent the mean plus standard deviation from 7 measurements.

Figure 2a shows the SERS signals measured from the PNI sensors while varying the UO_2_^2+^ concentration from 1 pM to 100 nM. The samples were prepared by a 1/10 serial dilution, and a blank sample solution was prepared as a control. In the blank sample solution, the PNI sensor provided a weak Cy5 SERS signal (black spectrum in Fig. 2a). In the 1 pM sample, the signal was significantly enhanced (red spectrum in Fig. 2a). Figure 2b shows the intensity of the Cy5 1580 cm^−1^ band plotted as a function of the UO_2_^2+^ concentration. The SERS signal from the PNI sensor increased as the UO_2_^2+^ concentration increased, saturating at a concentration of 10 nM. The inset in Fig. 2b shows the dynamic range of the PNI sensor for UO_2_^2+^ detection. The SERS signal intensity linearly increased within the UO_2_^2+^ concentration range of 1 pM to 10 nM. This wide dynamic range makes this technique advantageous for the quantitative detection of UO_2_^2+^. We estimated the detection limit of the PNI sensor to be 1 pM. This detection limit is approximately 1,000-fold lower than the limits of other SERS-based sensors^21^. Furthermore, the detection limit of this method is comparable to that of the most sensitive detection method, which is based on a resonance scattering spectral technique^15^.

**Figure 3:**
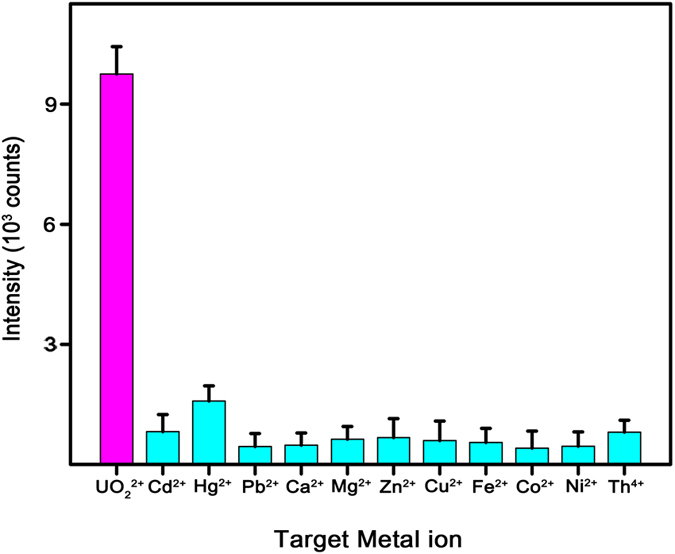
Selectivity of a PNI sensor for UO_2_^2+^ detection. The tested metal ions are shown on the *x* axis and their corresponding Cy5 1580 cm^−1^ band intensities are shown on the *y* axis. Strong SERS signals were observed only in the presence of UO_2_^2+^ (magenta bar) and weak SERS signals were observed in the presence of other metal ions (cyan bars). The data represent the mean plus standard deviation from 7 measurements.

To confirm the selectivity of a PNI sensor combined with a DNAzyme-cleaved reaction, SERS signals were observed from the PNI sensors after mixing DNAzyme with samples containing various metal ions (UO_2_^2+^, Cd^2+^, Hg^2+^, Pb^2+^, Ca^2+^, Mg^2+^, Zn^2+^, Cu^2+^, Fe^2+^, Co^2+^, Ni^2+^, and Th^4+^). The concentration of each metal ion was 10 nM. Figure 3 shows the intensity of the Cy5 1580 cm^−1^ band measured from the PNI sensors for detecting the various metal ions. Remarkable SERS signal was observed only in the presence of UO_2_^2+^ (magenta bar in Fig. 3), and no distinct SERS signal was observed in the presence of other metal ions (cyan bars in Fig. 3). These results indicate that the proposed detection method is highly specific for UO_2_^2+^.

**Figure 4:**
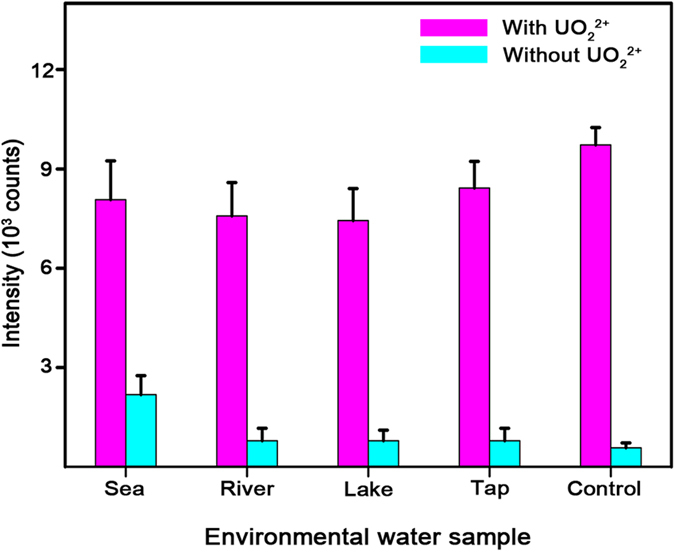
Applicability of a PNI sensor for UO_2_^2+^ detection in natural water. Natural water samples are shown on the *x* axis and their corresponding Cy5 1580 cm^−1^ band intensities are shown on the *y* axis. The magenta bars represent the natural water samples spiked with UO_2_^2+^ and the cyan bars represent the as-collected natural water samples. The spiked UO_2_^2+^ concentration was 10 nM. The strong SERS signals were observed only after the addition of UO_2_^2+^ into the natural water samples. The data represent the mean plus standard deviation from 7 measurements.

Finally, we examined the applicability of a PNI sensor combined with a DNAzyme-cleaved reaction to detect UO_2_^2+^ in natural water. The natural water samples were obtained from various environments, including a sea, a river, a lake, and a tap. Buffer solution was also prepared as a control. Figure 4 shows the intensities of the Cy5 1580 cm^−1^ band measured from the PNI sensors after mixing DNAzyme with the natural water samples. The magenta bars represent the natural water samples spiked with UO_2_^2+^ and the cyan bars represent the as-collected natural water samples. Strong SERS signals were observed only after the addition of UO_2_^2+^ into the natural water samples. Without UO_2_^2+^, SERS signals were rarely observed. Note that the DNAzyme is sensitive to ionic strength^56^. The DNAzyme is in the lock-and key mode at the ionic strength of 100 mM or higher^56^. In addition, the concentration of Mg^2+^ can affect the DNAzyme activity^56^. At the lower concentration of 2 mM, the activity is promoted. On the other hand, the activity is inhibited at the higher concentration of 2 mM. In this experiment, the DNAzyme solution and natural water sample were mixed with 9:1 volume ratio before the SERS measurement. The ionic strengths of the mixtures are calculated as 246.43 mM (sea), 131.81 mM (river), 134.72 mM (lake), and 132.07 mM (tap) according to the inductively coupled plasma-optical emission spectroscopy (ICP-OES) and ion chromatography (IC) data of natural water samples (Table S1 and S2 in supplementary information). The concentrations of Mg^2+^ in the mixtures are 1.31 mM (sea), 0.01 mM (river), 0.02 mM (lake), and 0.01 mM (tap). Since the ionic strengths of mixtures are higher than 100 mM and the concentrations of Mg^2+^ in the mixtures are below 2 mM, the PNI sensor combined with a DNAzyme-cleaved reaction can detect UO_2_^2+^ successfully even in natural water samples. The USA’s limit for UO_2_^2+^ in drinking water is 30 μg/L (approximately 100 nM), however, the long-term consumption and exposure to water that contains UO_2_^2+^, even at concentrations below this limit, can cause severe toxicity and serious diseases^57^. We anticipate that the present method can be employed for practical UO_2_^2+^ detection and aid in the prevention of environmental pollution and human diseases caused by UO_2_^2+^.

## Conclusion

We detected UO_2_^2+^ by combining an ultrasensitive PNI platform with a DNAzyme-cleaved reaction. The DNAzyme specifically reacted with UO_2_^2+^ and released a cleaved strand. The PNI sensor sensitively captured the cleaved strand, which enabled the detection of UO_2_^2+^. We quantitatively detected UO_2_^2+^ with an ultralow detection limit of 1 pM and high selectivity. Moreover, this method enables the detection of UO_2_^2+^ in various natural water sources, such as sea, lake, river, and tap. A PNI sensor that can precisely detect small quantities of UO_2_^2+^ in natural water is expected to reveal many environmental pollutants, and hence minimize damage to the human body caused by UO_2_^2+^ exposure.

## Methods

### Materials

Purified DNA was purchased from Bioneer (Daejeon, Korea). Hg(Ac)_2_, Mg(Ac)_2_, Ca(Ac)_2_, CrCl_2_, FeCl_2_, Co(Ac)_2_, NiCl_2_, CuCl_2_, Zn(Ac)_2_, Cd(Ac)_2_, Pb(Ac)_2_, UO_2_(Ac)_2_, Au powder (99.99%), and sodium dodecyl sulfate (SDS) were purchased from Sigma–Aldrich. Th(NO_3_)_4_ was purchased from Merck. Phosphate buffered saline (PBS) was purchased from Gibco. The natural water samples were collected from the west sea of South Korea, the Gap River, and the pond at KAIST. Samples were centrifuged to remove impurities.

### Preparation of DNAzyme

The sequence of the substrate strand is 5′-Cy5-TAATACACTCACTAT(rA)GGAAGAGATGGACGTG-3′, and the sequence of the enzyme strand is 5′-CACGTCCATCTCTGCAGTCGGGTAGTTAAACCGACCTTCAGACATAGTGAGT-3′. The sequence of the capture strand is 5′-ATAGTGAGTGTATTA-SH-3′. The substrate and enzyme strands were mixed in a 1× PBS solution (pH 7.4) at the same molar concentration (10 μM each). The hybrid solutions were heated at 95 °C for 10 min and slowly cooled to room temperature.

### Synthesis of single-crystalline Au NWs

Single-crystalline Au NWs were synthesized on a *c*-cut sapphire substrate in a horizontal quartz tube furnace system following the chemical vapor transport method described in a previous report^54^. An alumina boat containing an Au powder was positioned directly below the heat source. The sapphire substrate was placed a few centimeters downstream from the alumina boat. The heating zone was brought to 1100 °C while the chamber pressure was maintained at 3–5 Torr. Ar gas flowing at 100 sccm was used to transport the Au vapor. Au NWs were grown on the sapphire substrate over a 1 h period.

### Preparation of PNI sensors

Au film substrates were prepared on Si substrates by electron beam-assisted deposition of a 10 nm thick film of Cr followed by a 300 nm thick film of Au. The prepared Au films were SERS-inactive by themselves^24^. The Au films were then cut to 1 cm^2^ for PNI sensor fabrication. To prepare the capture probe DNA-attached Au NWs, as-synthesized NWs were incubated with 5 mM captured DNA in 1 M KH_2_PO_4_ buffer (pH 6.75) at room temperature for 12 h. Next, the Au NWs were rinsed with a 0.2% (w/v) SDS solution for 5 min. The capture probe DNA-attached Au NWs were then transferred onto Au films by a simple attachment and detachment process^30^. Briefly, the NW-grown *c*-cut sapphire substrates were inverted onto Au film substrates containing medium. Before both substrates were overlapped, a drop of distilled water was applied as a lubricant. The sapphire substrates were then pushed gently and, after a few seconds, detached. After the attachment and detachment process, the remaining water was dried under flowing N_2_ gas.

### Detection of UO_2_^2+^ using PNI sensors combined with a DNAzyme-cleaved reaction

DNAzyme in 1× PBS (pH 7.4) with RNase-free water was mixed with sample solutions containing 0.27% (w/v) HNO_3_ and incubated for 10 min. The pH of the mixed solution was maintained at 5.49 and its ionic strength was 146.43 mM. To stop the enzymatic reaction by shifting the pH of the mixture, 0.1 mM Tris-acetate solution was added. Then, the mixture was dropped onto the PNI sensor and allowed to stand for 2 h. To remove excess DNA, the sensor was rinsed with a 0.2% (w/v) SDS solution for 5 min and then rinsed twice with distilled deionized water.

### Instrumentation

SERS spectra were measured using a micro-Raman system on an Olympus BX41 microscope. A 633 nm He/Ne laser (Melles Griot) was used as an excitation source, and the laser was focused on samples through a 100× objective (NA = 0.7, Mitutoyo). The laser power directed at the sample was 0.4 mW. The SERS signals were recorded with a thermodynamically cooled electron-multiplying charge-coupled device (Andor) mounted on the spectrometer with a 1200 groove/mm grating. The acquisition time for all SERS spectra was 60 s. A holographic notch filter was used to reject laser light. The concentration of metal ion in natural water samples was analyzed by inductively coupled plasma-optical emission spectroscopy (ICP-OES 720, Agilent) and ion chromatography (881 Compact IC pro, Metrohm Ltd.).

## Additional Information

**How to cite this article**: Gwak, R. *et al.* Precisely Determining Ultralow level UO_2_^2+^ in Natural Water with Plasmonic Nanowire Interstice Sensor. *Sci. Rep.* **6**, 19646; doi: 10.1038/srep19646 (2016).

## Supplementary Material

Supplementary Information
