# Supplementary material for: Precisely Determining Ultralow level UO22+ in Natural Water with Plasmonic Nanowire Interstice Sensor
Source: Sci Rep. 2016 Jan 21;6:19646. doi: 10.1038/srep19646 (PMC4726367; doi:10.1038/srep19646)
Supplement: Supplementary Information [file srep19646-s1.doc]

**Supplementary Information**

**Precisely Determining Ultralow level UO_2_^2+^ in Natural Water with Plasmonic Nanowire Interstice Sensor**

Raekeun Gwak^1,+^, Hongki Kim^1,+^, Seung Min Yoo^2^, Sang Yup Lee^2^, Gyoung-Ja Lee^3^, Chang-Kyu Rhee^3^, Taejoon Kang^4,5,*^, and Bongsoo Kim^1,*^

^1^KAIST, Department of Chemistry, Daejeon 34141, Korea

^*^bongsoo@kaist.ac.kr

^2^KAIST, Department of Chemical and Biomolecular Engineering, Daejeon 34141, Korea

^3^KAERI, Nuclear Materials Development division, Daejeon 34057, Korea

^4^KRIBB, BioNanotechnology Research Center and BioNano Health Guard Research Center, Daejeon 34141, Korea

^5^UST, ​Major of Nanobiotechnology and Bioinformatics, Daejeon 34113, Korea

^*^kangtaejoon@kribb.re.kr

**Supplementary Table S1.** ICP-OES data of natural water samples.

**Supplementary Table S2.** IC data of natural water samples.

**Table S1.** **ICP-OES data of natural water samples.**

| Sample | Na (mM) | Mg (mM) | P (mM) | S (mM) | K (mM) | Ca (mM) |
| --- | --- | --- | --- | --- | --- | --- |
| River | 0.398 | 0.104 | 0 | 0.121 | 0.152 | 0.058 |
| Lake | 0.576 | 0.215 | 0 | 0.096 | 0.058 | 0.075 |
| Tap | 0.346 | 0.139 | 0 | 0.129 | 0.071 | 0.048 |
| Sea | 289.262 | 13.069 | 0 | 7.538 | 3.371 | 0.344 |

**Table S2.** **IC data of natural water samples.**

| Sample | Cl^-^ (mM) | SO_4_^2-^ (mM) |
| --- | --- | --- |
| River | 0.305 | 0.127 |
| Lake | 0.646 | 6.724 |
| Tap | 0.644 | 0.244 |
| Sea | 519.915 | 34.365 |
